# Mucormycosis originated total maxillary and cranial base osteonecrosis: a possible misdiagnosis to malignancy

**DOI:** 10.1186/s12903-021-01411-8

**Published:** 2021-02-12

**Authors:** Young Long Park, Sura Cho, Jin-Woo Kim

**Affiliations:** Department of Oral and Maxillofacial Surgery, School of Medicine, Ewha Womans University Medical Center, Mokdong Hospital, Anyangcheon-ro 1071, Yangcheon-gu, Seoul, 158-710 Korea

**Keywords:** Skull base, Osteomyelitis, Mycoses, Osteonecrosis, Mucormycosis, Neoplasms

## Abstract

**Background:**

This is a case of mucormycosis originated osteonecrosis of the maxilla extended to the cranial base, initially suspected of malignancy. The patient was first suspected with osteolytic sarcomatous lesion but was later diagnosed with total maxillary necrosis and cranial base through biopsy-proven invasive mucormycosis.

**Case presentation:**

A 71-year-old male was presented with unknown total maxillary osteonecrosis. CT and MRI results showed extensive osteolytic change with bone destruction of the cranial base, and PET-CT showed irregular hypermetabolic lesion in the area suspected of malignancy. The first biopsy results only presented tissue inflammation. Thus, several further endoscopic biopsy were performed through posterior pharyngeal wall. The patient was eventually diagnosed with mucormycosis and associated osteomyelitis with subsequent bone necrosis. With confirmed diagnosis, partial maxillectomy of the necrosed bone was performed under general anesthesia. At the 4 week follow-up, the patient showed full mucosal healing and no recurrence or aggravation of the maxilla and cranial base lesion was observed.

**Conclusions:**

Accurate diagnosis of atypical symptoms, timely diagnosis, and proper combination therapy of surgical intervention, antifungal agent, and antibiotic use for skull base osteomyelitis are all critical for proper treatment planning. In addition, biopsy and CT scans are essential in differentiating osteonecrosis from malignancy.

## Background

Jaw bone necrosis has been known to be associated with various conditions including trauma, radiation therapy, infections, antiresorptive medications, etc. The anatomical uniqueness of the jaw bone frequently confuses surgeons with differential diagnosis of osteonecrosis of the jaw.

Skull base osteomyelitis (SBO) is a rare disease with both diagnostic and therapeutic challenges. There have been few reported cases of chronic invasive mucormycotic sinusitis causing skull base osteomyelitis [1], but skull base involvement is rare and its onset is relatively late. Mucormycosis more frequently results in sinonasal, orbital, and deep facial soft-tissue infiltration, providing an ideal environment for bacterial growth. It involves intracranial complications such as cavernous sinus involvement, cerebral abscesses, and infarcts. The late occurrence of bony involvement is explained by the angioinvasive nature of the fungus and deep penetration of the infection through perivascular channels that precede bony destruction [2]. Fungal infection originated osteomyelitis, which preferentially invades the bone over other areas, is very rare.

Clinicians face many challenges in diagnosing invasive fungal infection. Non-specific symptoms present difficulties in definitive early diagnosis. Radiographic images may be useful for early diagnosis, but it lacks accuracy and is difficult to differentiate from malignancy.

The authors report a case of a patient who was first suspected of malignancy but was later diagnosed with total maxillary necrosis and skull base osteomyelitis (SBO) through radiograph readings and biopsy-proven invasive mucormycosis.

## Case presentation

**Fig. 1:**
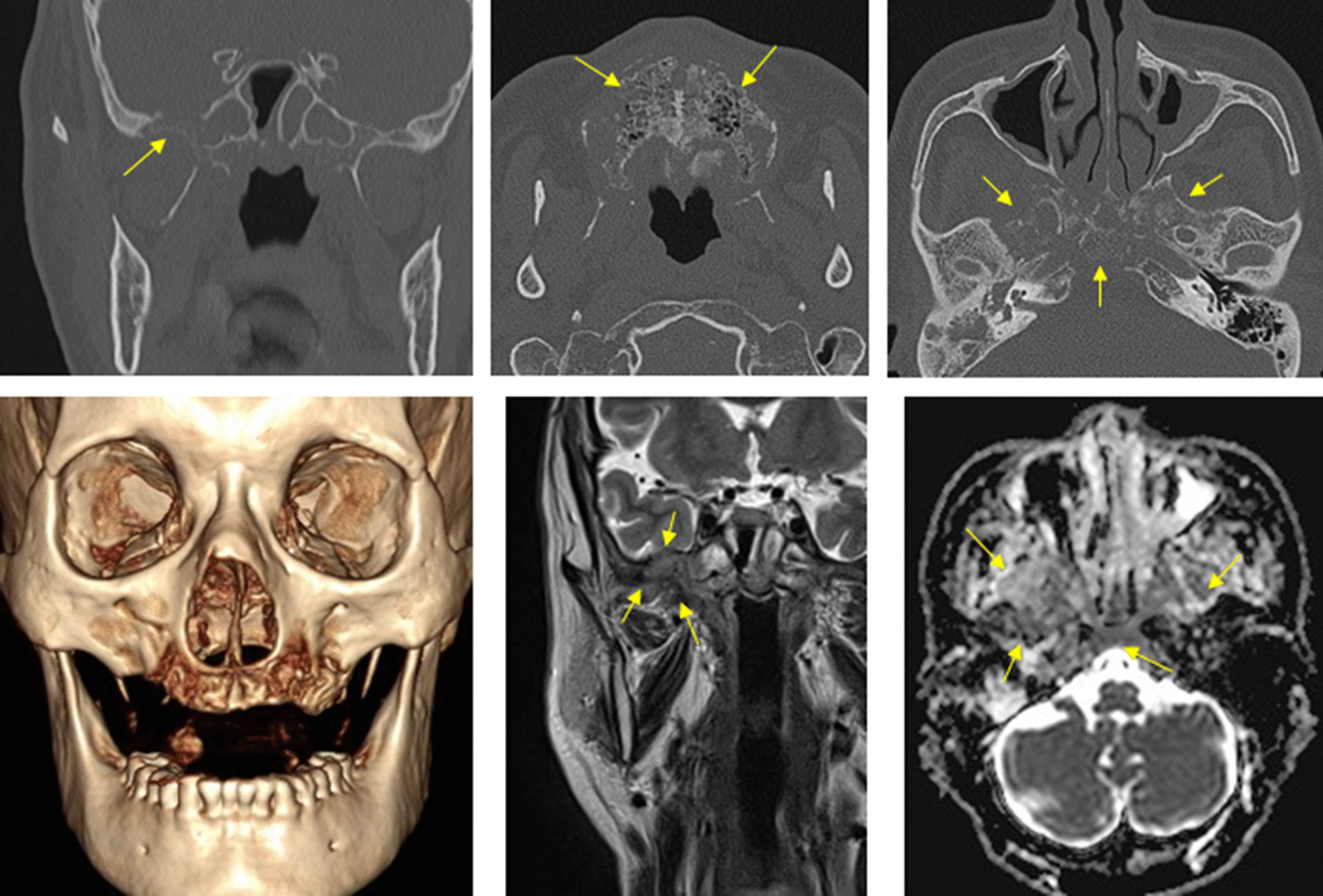
CT scan and MRI showing maxilla osteonecrosis. There is extensive osteolytic change with bone destruction of the central skull base including sphenoid body, sinus, right greater wing, and pterygoid process

**Fig. 2:**
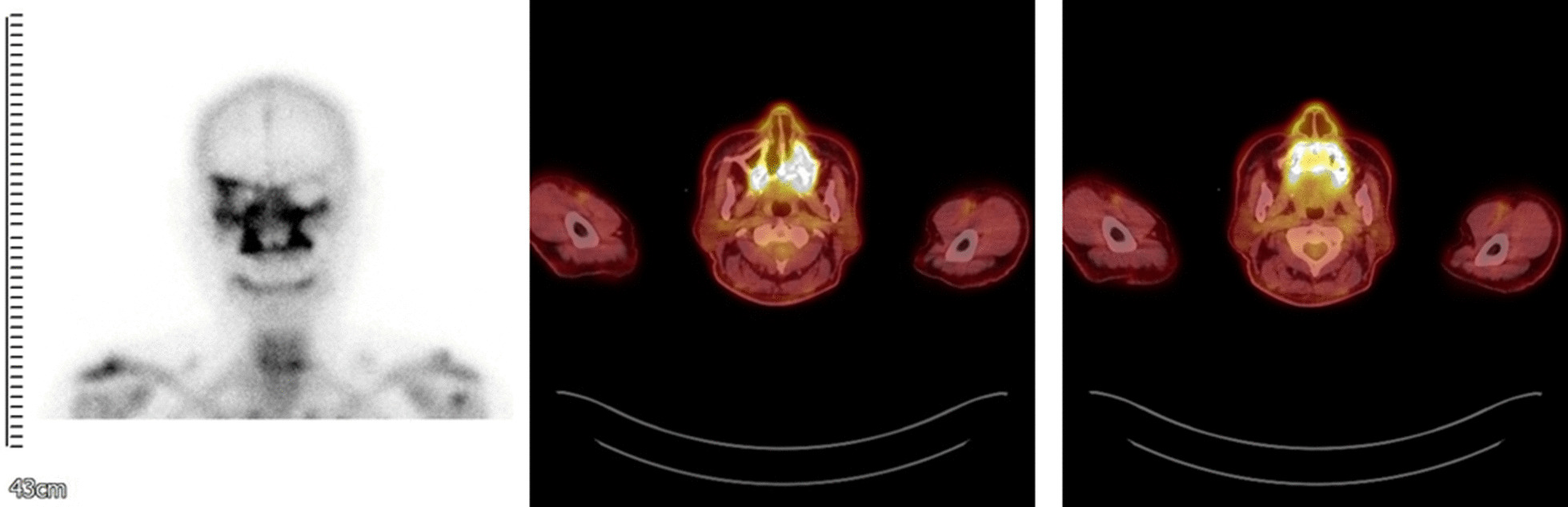
Bone scan showing active bone lesion around both maxillary and right whole skull base PET-CTs also showing irregular hypermetabolic lesion involving skull base, posterior nasopharyngeal wall, and both maxillary area

A 71-year-old male was presented as a referral from a local dental clinic for unknown total maxillary osteonecrosis. The patient had a medical history of controlled hypertension and diabetes mellitus. After radiographic and clinical evaluation, he was shown with extensive osteonecrosis of the entire maxilla (Fig. 1) and was hospitalized under the department of oral and maxillofacial surgery. He was immediately prescribed with prophylactic antibiotics in preparation for surgery. On admission, the patient had unremarkable vital signs and no abnormalities in neurologic examination. Laboratory studies showed normal white blood cell count (7080 cells/μl, normal erythrocyte sedimentation rate (14 mm/h), and normal serum C-reactive protein level of 0.22 mg/dL. Necessary radiographic images including computed tomography, MRI, and bone scan were taken (Fig. 1). Bone scan showed active bone lesion around both maxillary and was shown to have extended to the upper right orbit (Fig. 2). The readings of CT and MRI showed total necrosis of the maxilla, and osteomyelitis was extended to the sphenoid body, sinus, right greater wing, pterygoid process, and the clivus. PET-CT showed irregular hypermetabolic lesion involving the skull base, posterior nasopharyngeal wall, and both maxillary areas, indicating possible malignancy (Fig. 2). Tentative initial treatment plan included total maxillectomy with reconstruction, but radiologic exam indicated a need for pathologic confirmation before proceeding with any definitive treatment.

One week following admission, an incisional biopsy of the necrotic bone in the posterior right and left maxilla was first performed under local anesthesia. However, considering the risk of invasiveness in skull base biopsy, the department of neurosurgery deferred a second biopsy and instead, suggested to follow up with the patient after 3 months. The histopathological analyses of the posterior right maxilla sequestrum, right interior turbinate, and nasopharynx punch biopsy showed non-specific inflammation of the granulation tissue unlike the CT readings that appeared as lesions. The diagnosis was uncertain at this point whether the infected area was a malignant tumor or a benign disease. Although the department of neurosurgery recommend waiting for follow up after the first biopsy, considering the mortality and high morbidity of the suspicious area, the authors decided to immediately perform a deeper second biopsy after the results of the first biopsy was obtained. Further endoscopic biopsy was performed in the maxillary sinus posterior inferior wall under general anesthesia and the biopsy specimen involved the nasopharynx, the sphenoid bone, and the pterygoid bone.

**Fig. 3:**
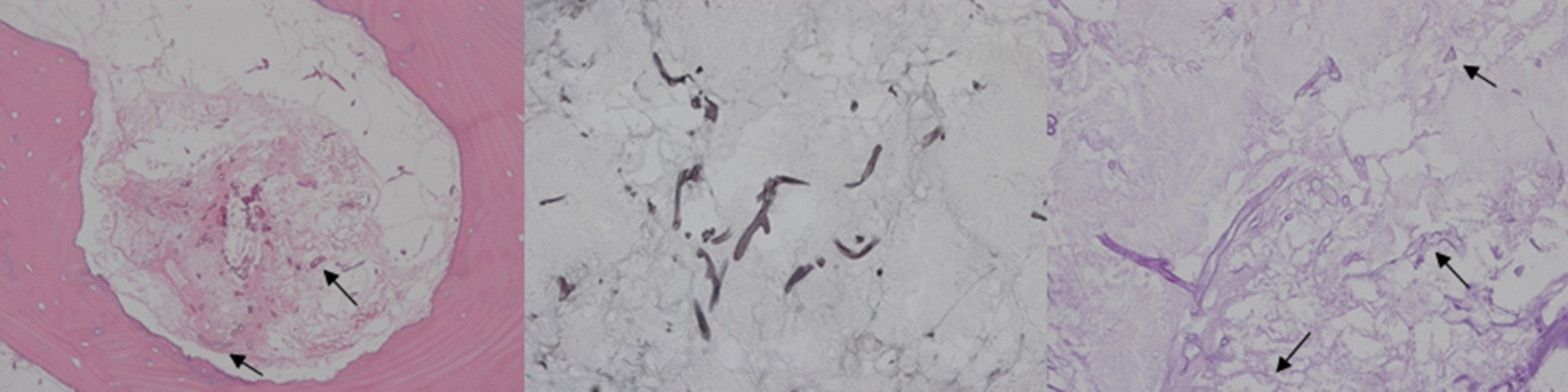
Histologic demonstration of mucormycosis lesion hematoxylin and eosin stain (×200) Grocott methenamine-silver (GMS) staining (×400) Periodic acid-Schiff (PAS) staining (×200)

**Fig. 4:**
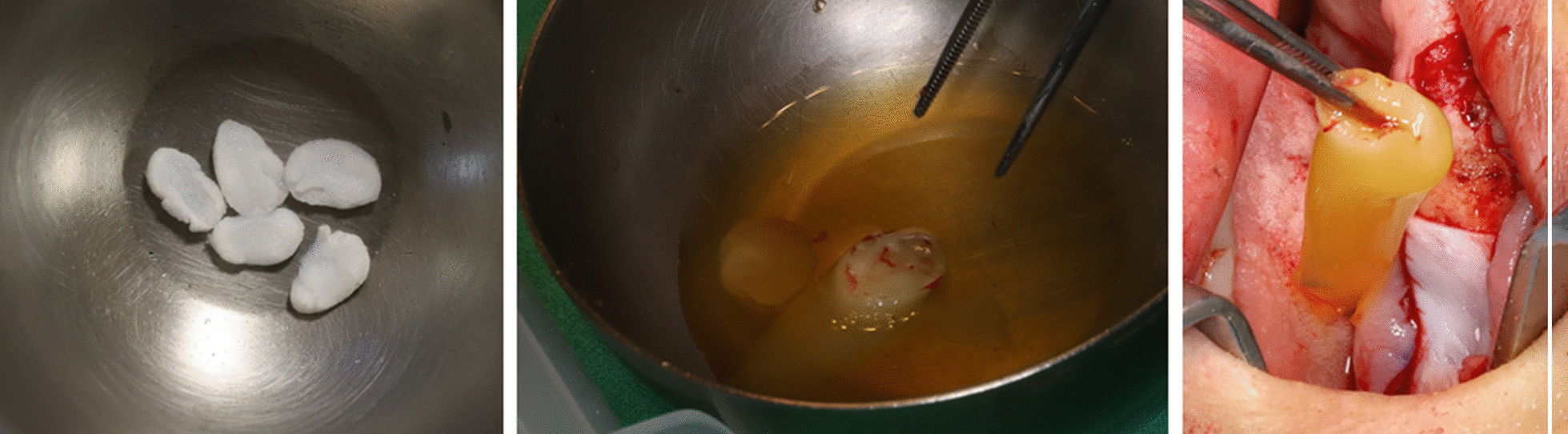
Preparation of recombinant human bone morphogenetic protein-2 (rhBMP-2) and Collagen sponge, which were soaked in the recombinant human bone morphogenetic protein 2 solution. Leukocyte-rich and platelet-rich fibrin was obtained and rhBMP-2 is first brought into direct contact with the bone surface, then L-PRF is applied

**Fig. 5:**
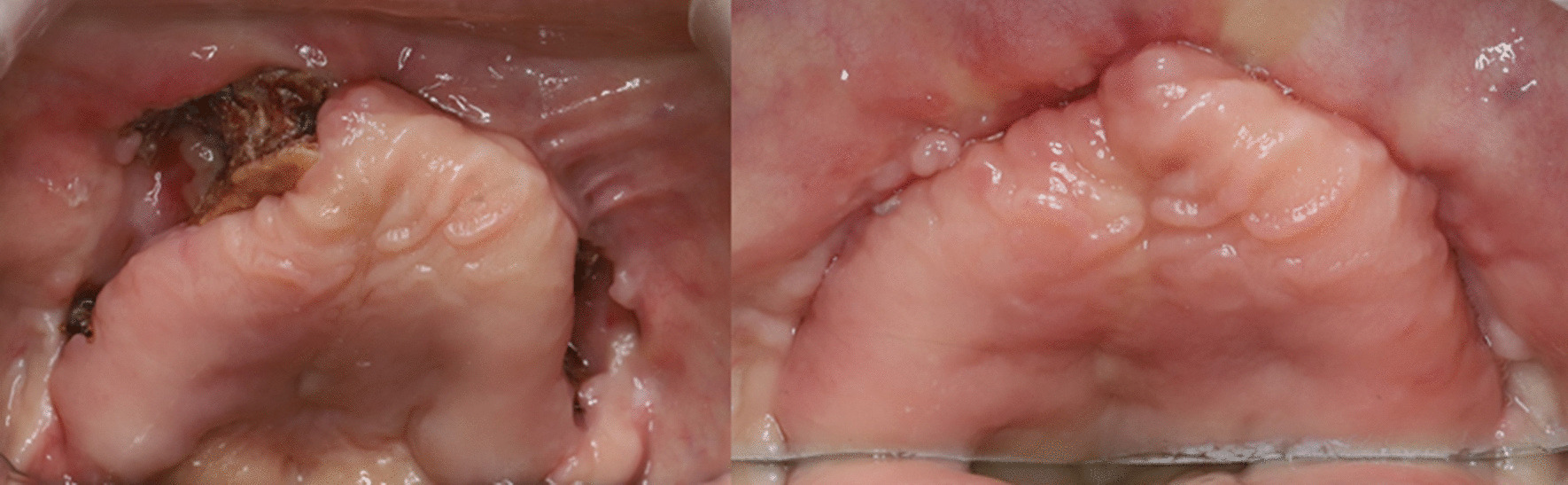
Clinical image showing gingival dehiscence and osteonecrosis and postoperative 4 week follow-up showing full mucosal healing status

**Fig. 6.**
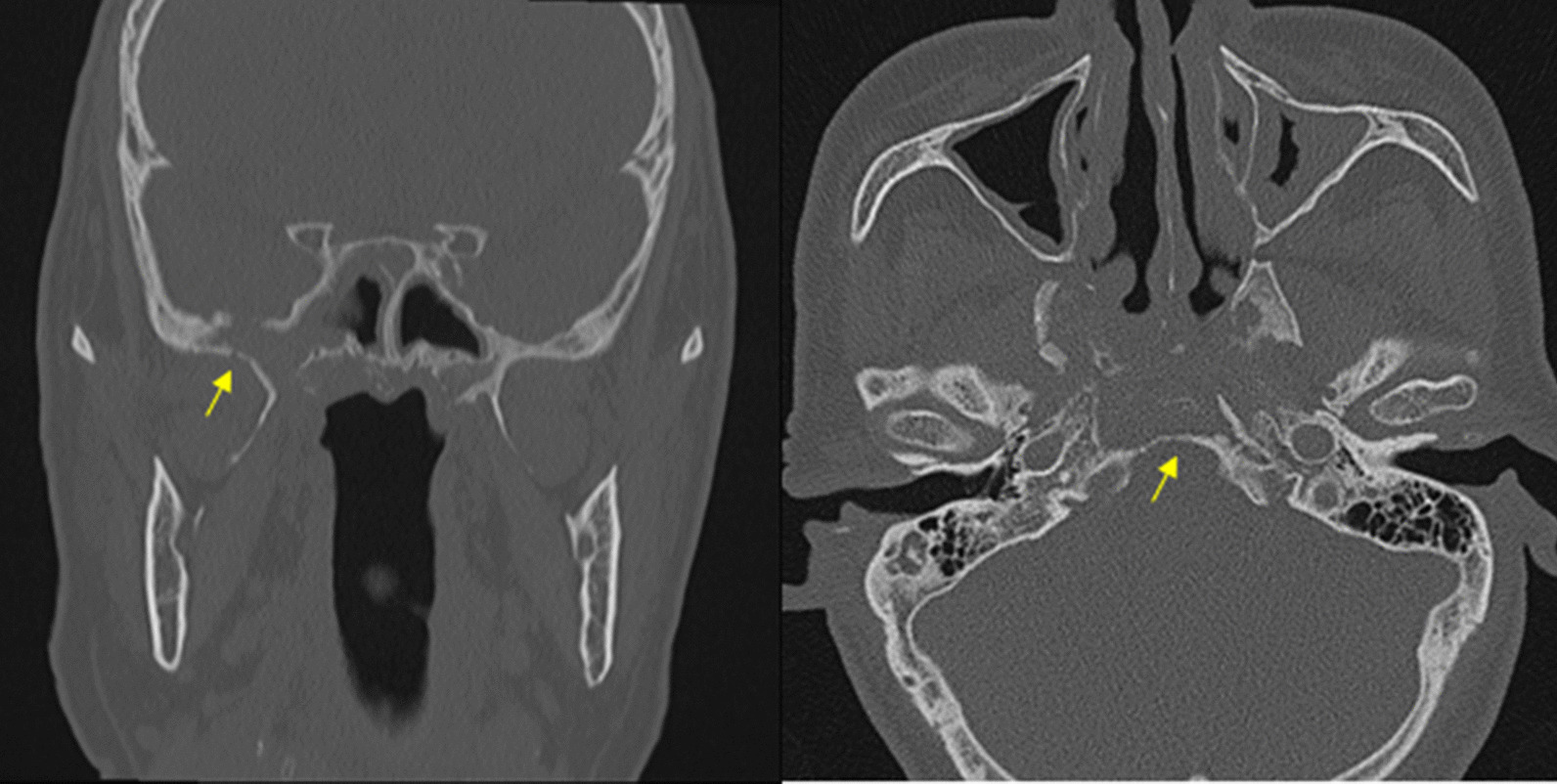
4 Months follow-up CT showing improved osteolytic change

The authors did not find any evidences of malignancy from both biopsy results, but instead, several degenerated fungal hyphae was found in the right pterygoid region of the sphenoid bone. The patient was diagnosed with mucormycosis and associated osteomyelitis with subsequent bone necrosis (Fig. 3). 350 mg of anti-fungal agent, Amphotericin B was administered once a day via intravenous injection. Patient lab testing results and symptoms began to improve although previous administration of 3rd generation cephalosporin was ineffective. The 2-month follow-up CT and MRI results showed improved osteolytic change in the sphenoidal bone of cranial base, especially around the right greater wing and the clivus. Under confirmed diagnosis of fungal infection originated osteomyelitis and bone necrosis, we followed the protocol with preoperative and postoperative antibiotic support, necrotic bone removal and debridement in conjunction with leukocyte-rich and platelet-rich fibrin (L-PRF), and placement of recombinant human bone morphogenic protein (rhBMP-2). To make L-PRF, the authors collected 10–20 ml of peripheral blood. Blood samples were collected in an anticoagulant-free 8.5 ml tube and immediately centrifuged. Centrifugation should be performed at 3000 rpm for 10 min. Before centrifugation, a trained assistant should check to prevent coagulation of the cascade and to naturally transform the fibrin matrix during the centrifugation process. After centrifugation, L-PRF can be obtained at the center of the tube, and red blood cells at the bottom of the remaining tubes and acellular plasma at the top were not used. rhBMP-2 can be made using a commercial kit (Novosis; Daewoong Pharma, Seoul, Republic) containing 0.5 mL of rhBMP-2 solution and hydroxyapatite. The collagen sponge can be used as a rhBMP-2 carrier, which is split into thin and rounded pieces and immersed in the rhBMP-2 solution. The kit's hydroxyapatite is not used [3, 4]. The use of rhBMP and L-PRF was approved by institutional review board (EUMC 14-20A-02). Bony sequestra and granulation tissue were removed by surgical curettes until fresh bleeding from bone was confirmed. Rotary instruments were used to smoothen any remaining bony margins [5]. rhBMP-2 is first brought into direct contact with the bone surface, then L-PRF is applied (Fig. 4). The patient showed full mucosal healing status at the 4 week follow-up (Fig. 5). The anti-fungal agent was changed from an IV injection to a PO medication (Posaconazole 400 mg, twice daily) and we confirmed stabilized symptoms (Fig. 6). Future reconstruction treatment included the implementation of implant-supported overdenture with bone graft.

## Discussion and conclusions

Several testing were necessary before accurate diagnosis was made on this patient. This case particularly emphasized the importance of timely diagnosis and proper planning of combination therapy between surgical intervention and the use of antifungal agent and antibiotics for CSBO. Accurate diagnosis and proper treatment planning at proper timing may prevent extreme outcomes such as mortality and also minimize permanent neurologic deficits.

SBO is a rare disease and mostly caused by continuous ear infections [6, 7]. It usually occurs in diabetics and immunosuppressive patients, especially in those who have poor healing of necrotizing otitis externa [8]. SBO rarely results from sinonasal infections, which include microorganisms such as Aspergillus, Pseudomonas, Salmonella, and Staphylococcus. Rather, complex infections of paranasal sinusitis related to osteomyelitis tend to involve cancellous bone in cranial reservoirs such as the frontal bones rather than the skull base. Mucormycotic SBO is a rare disease and is characterized by its late diagnosis.

Rhinocerebral mucormycosis is characterized by acute, incurable diseases and often fatal opportunistic infections [9]. When the fungus enters the nasal and sinus cavity, it can cause necrotizing vasculitis in the nose and sinus, infecting an immunosuppressed or diabetic host, which can rapidly progress to orbit, deep faces, and skull cavity [10]. This is caused by perivascular, perineural, or direct soft-tissue invasion of the fungi, which in turn causes suppurative arteritis, vascular thrombosis, and infarction of the surrounding tissues.

Skull base osteomyelitis and bone necrosis from sinonasal mucormycosis is often has no symptoms despite deep penetration of the disease, and even if there are symptoms, occurs later [9, 11]. This is thought to be due to the vascular permeability of fungi and their tendency to expand into the soft tissues of the orbit, deep face, and brain through blood vessels penetrating through the partitions at the base of the skull. The treatment of chronic rhinocherebral mucormycosis is not well known, but a few include: surgical treatment of the lesion, administration of prolonged high-dose systemic amphotericin B treatment, correction of the metabolic disorder, and hyperbaric oxygen therapy.

Simultaneous Application of Bone Morphogenetic Protein-2 and Platelet-Rich Fibrin in osteonecrosis patients is effective in osteonecrosis of jaw patients. There have been studies on the use of bone morphogenetic protein 2 (BMP-2) for the treatment of osteonecrosis to increase bone remodeling. BMP-2, a member of the transforming growth factor β super family, has been widely used in the treatment of bone defects due to its osteoinductivity. Oversuppression of bone remodeling is considered one of the etiologies of osteonecrosis. Therefore, BMP-2 was thought to have potential reversing effect on remodeling-inhibited bone, which enhances bone remodeling in osteonecrosis. The application of platelet-rich plasma was attempted and showed good results in osteonecrosis healing. The rationale for the use of platelet concentrate for osteonecrosis is based on the role of growth factors in promoting and stimulating regeneration of soft and hard tissues. For the delivery of BMP, fibrin is known to support matrix for BMP, and the fibrin matrix associated with BMP has vascular nutrition, hemostasis, and osseous conductive properties. It is thought that BMP within a fibrin matrix such as PRF stimulates osseous regeneration as well as soft tissue healing, thereby contributing to the regeneration of hard tissue [3–5].

In the case of this patient, there were several factors that made accurate diagnosis difficult. The rarity of fungal infection originated osteonecrosis made it a difficult first diagnostic option. At the patient’s initial visit, the symptoms appeared to be necrosis of the maxilla, thus his medical history data was collected and simple radiographs were taken. When the bone scan showed extensive bone necrosis around both maxillary and upper right orbit, we performed CT, MRI, and PET-CT scans for the possibility of malignancy over osteonecrosis. When malignancy was first suspected from the radiograph readings, we considered operating a cancer surgery over ONJ. We could not exclude the possibility of malignancy after the readings of PET-CT. At the time of the first PET-CT scan, the maximum Standard uptake value (SUV) was 7.9 in the clivus, sphenoid bones, posterior nasopharyngeal wall, bilateral maxilla, and alveolar process of the skull base. K.A. Higgins et al. suggest that pretreatment SUV is a prognostic variable in head and neck cancer and increased SUV in the primary tumor is associated with decreased disease free survival. Additionally, SUV provides a better picture of the overall tumor metabolic activity [12]. An SUV of < 2.5 is generally considered to be indicative of a benign lesion, an SUV of 2.5 or higher is generally considered to be indicative of malignant tissue [13–15]]. With high SUV, we had to first doubt malignancy, thus prepared for a second biopsy operation under general anesthesia as soon as notifying the patient of possible malignancy. This case was difficult to discern the difference between malignancy and fungal infection through radiographic images including computed tomography, MRI, and PET-CT. A deep biopsy was needed for a more accurate diagnosis. With endoscopic biopsy results, we were able to fully confirm mucormycosis and Klebsiella pneumoniae infection.

Accurate diagnosis of a patient’s non-typical symptom is required for proper treatment planning. Early detection and proper planning of combination therapy between surgical intervention and use of antifungal agent and antibiotics is particularly important for CSBO. Additionally, biopsy and CT scans are essential in differentiating osteonecrosis from malignancies.

## Data Availability

The data that support the findings of this study are available from the corresponding author, JW Kim, upon reasonable request.

## Abbreviations

SBO: Skull base osteomyelitis
CSBO: Central skull base osteomyelitis
BMP: Bone morphogenic protein
PRF: Platelet rich fibrin
ONJ: Osteonecrosis of jaw
SUV: Standard uptake value
